# Regional brain dysfunction patterns associated with rapid eye movement sleep behavior disorder and visual hallucinations in Parkinson’s disease: a resting-state fMRI study with exploratory ROI-based factorial analysis

**DOI:** 10.3389/fneur.2026.1858348

**Published:** 2026-06-24

**Authors:** Lifang She, Xiong Wei, Hongyang Cai, Liuchen Zhou, Zonghong Li, Yang Pan

**Affiliations:** 1 Department of Geriatrics, The Affiliated Brain Hospital of Nanjing Medical University, Nanjing, China; 2 School of Computer Science, Wuhan University, Wuhan, Hubei, China; 3 Department of Image, Affiliated Brain Hospital of Nanjing Medical University, Nanjing, China; 4 Department of Neurology, Zhongnan Hospital of Wuhan University, Wuhan, Hubei, China

**Keywords:** freezing of gait, Parkinson’s disease, rapid eye movement sleep behavior disorder, resting-state fMRI, visual hallucinations

## Abstract

**Background:**

Rapid eye movement sleep behavior disorder (RBD) and visual hallucinations (VH) are prognostically relevant non-motor symptoms in Parkinson’s disease (PD), but their combined effects on local brain dysfunction remain unclear.

**Objective:**

To characterize regional brain dysfunction patterns associated with RBD and VH in PD and to explore candidate-region symptom-related effects within regions showing overall between-group differences.

**Methods:**

In this cross-sectional study, 96 patients with PD were divided into four groups according to the presence or absence of RBD and VH (24 per group). Resting-state functional MRI was analyzed using amplitude of low-frequency fluctuations (ALFF) and regional homogeneity (ReHo). Whole-brain four-group analyses were first used to identify regions with overall between-group differences, followed by exploratory ROI-based 2 × 2 factorial analyses within candidate regions. Additional whole-brain voxel-wise 2 × 2 factorial analyses were performed as supplementary analyses. Correlations between imaging indices and clinical scales were also examined.

**Results:**

Patients with both RBD and VH showed the greatest clinical burden and worse cognitive performance. Whole-brain analyses revealed abnormalities in frontal, temporal, cerebellar, supplementary motor, and precuneus regions. Exploratory candidate-region analyses with Benjamini–Hochberg FDR correction showed RBD-related patterns in precuneus ReHo, cerebellar lobule VIII ReHo, and SMA ALFF; VH-related patterns in OFC ReHo, precuneus ReHo, cerebellar Crus I ReHo, SMA ALFF, and temporal pole ALFF; and interaction-like patterns in OFC ReHo and temporal pole ALFF. These ROI-based findings were interpreted as *post hoc* exploratory results rather than independent confirmatory evidence. Imaging abnormalities were correlated with RBD severity, freezing of gait, hallucination burden, and cognition.

**Conclusion:**

Coexisting RBD and VH may identify a clinically more severe PD subtype associated with regional abnormalities involving cerebellar, motor, default mode, and association-related regions. Symptom-related main and interaction patterns should be interpreted as candidate-region exploratory findings requiring further confirmation in larger studies.

## 1 Introduction

Parkinson’s disease (PD) is a neurodegenerative disorder with marked clinical heterogeneity (1). As the disease progresses, non-motor symptoms (NMS) often have a greater impact on quality of life and prognosis than motor symptoms (2, 3). Among these, rapid eye movement sleep behavior disorder (RBD) and visual hallucinations (VH) are both regarded as prognostically relevant biological markers (4–6). Previous studies have shown that RBD is not only a prodromal manifestation of α-synucleinopathy, but also predicts a more rapid decline in motor function and a higher risk of cognitive impairment in patients with PD (7, 8). Similarly, VH are a hallmark psychiatric manifestation in PD and are closely associated with impairments in attention, executive function, and visuospatial processing. They are also considered an important predictor of PD dementia and adverse long-term outcomes (9, 10).

RBD and VH do not occur independently in PD. Epidemiological studies have shown that PD patients with RBD are at a substantially increased risk of developing VH (11, 12). Patients with both RBD and VH may represent a more severe clinical subtype, characterized by faster cognitive decline and greater brain atrophy (13, 14). These observations raise the possibility that coexisting RBD and VH reflect broader network dysfunction, rather than the simple coexistence of two non-motor symptoms.

Current neuroimaging studies in PD have examined RBD and VH separately. In VH, the widely accepted hypothesis posits that its core mechanism stems from decoupling between insufficient activation of the dorsal attentional network (DAN) and excessive activation of the default mode network (DMN) (15–17). In contrast, although RBD has traditionally been associated with brainstem pathology such as the sublaterodorsal nucleus (18, 19), more recent neuroimaging studies have shown that functional alterations in patients with RBD extend to the cerebellum, thalamus, and sensorimotor cortex (20, 21). Although these separate lines of research help identify symptom-related neural correlates, they do not clarify whether the two symptoms exert interactive rather than merely additive effects on brain function.

Therefore, this study classified PD patients according to the presence or absence of RBD and VH and combined amplitude of low-frequency fluctuations (ALFF) and regional homogeneity (ReHo) to investigate local brain dysfunction across four clinically defined subgroups. Whole-brain analyses were first used to identify regions showing overall between-group differences, followed by exploratory ROI-based factorial analyses to further characterize the relative contributions of RBD and VH within these candidate regions. This study aimed to characterize regional brain dysfunction patterns associated with RBD and VH in PD and to explore candidate-region symptom-related effects across four clinically defined subgroups.

## 2 Methods

### 2.1 Participants

This study enrolled a total of 96 PD patients, who were divided into four groups: RBD with VH group (PD-RBD^+^VH^+^), RBD without VH group (PD-RBD^+^VH^−^), non-RBD with VH group (PD-RBD^−^VH^+^), and non-RBD without VH group (PD-RBD^−^VH^−^), with 24 cases in each group. All participants in this study were recruited from the Affiliated Brain Hospital of Nanjing Medical University. Written informed consent was obtained from all participants. The study protocol was approved by the Ethics Committee of the Affiliated Brain Hospital of Nanjing Medical University (Ethics Approval No: 2022-KY054-01), and all procedures were conducted in accordance with the Declaration of Helsinki.

RBD status was determined using a structured clinical assessment that incorporated patient history, family report when available, and the Rapid Eye Movement Sleep Behavior Disorder Screening Questionnaire (RBDSQ). Participants with clinically suggestive dream-enactment behaviors together with an RBDSQ score ≥ 6 were classified as probable RBD-positive (22, 23), whereas those without suggestive clinical history were classified as RBD-negative. VH status was determined using a structured clinical interview, supplemented by family report when available, together with assessment using the University of Miami Parkinson’s Disease Hallucinations Questionnaire (UM-PDHQ) (24). Participants were classified as VH-positive when recurrent visual hallucinations were identified during clinical interview and supported by UM-PDHQ findings. Those without a history of visual hallucinations were classified as VH-negative (25–27). Grouping was completed based on the presence or absence of RBD and VH. Because polysomnography was not available for all participants, RBD status was classified as probable RBD based on clinical history, family report, and RBDSQ score. Similarly, VH status was determined using structured clinical interview and UM-PDHQ assessment rather than a formal structured psychiatric diagnostic interview.

Exclusion criteria were as follows: (1) atypical or secondary parkinsonism; (2) major neurological disorders other than PD; (3) severe cognitive impairment or inability to complete clinical assessment; (4) current delirium, acute confusional state, or other clinically unstable neuropsychiatric condition; (5) a history of major psychiatric disorders unrelated to PD; (6) severe visual or auditory impairment that could interfere with symptom evaluation; (7) severe systemic disease affecting brain function or MRI participation; (8) recent major medication adjustment; and (9) contraindications to MRI.

### 2.2 Clinical evaluation

All participants underwent standardized clinical assessment on the same day as MRI acquisition under their usual stable medication regimen. To reduce the influence of short-term clinical fluctuation, both clinical evaluation and MRI scanning were performed under the same medication condition. Antiparkinsonian medication burden was estimated using levodopa equivalent daily dose (LEDD).

Clinical assessments were performed by two experienced neurologists, and any discrepancies were resolved by consensus. The Unified Parkinson’s Disease Rating Scale (UPDRS) was used to assess the severity of overall motor and non-motor symptoms in patients; The Hamilton Depression Rating Scale (HAMD) and Hamilton Anxiety Rating Scale (HAMA) were used to assess the patients’ depressive and anxiety states, respectively; The Montreal Cognitive Assessment (MoCA) was used to evaluate overall cognitive function; The Freezing of Gait Questionnaire (FOGQ) was used to assess the severity of freezing gait in Parkinson’s disease patients and its impact on daily ambulation function; The RBDSQ was used to assess symptoms related to rapid eye movement sleep behavior disorder; The presence and severity of visual hallucinations were assessed using the UM-PDHQ; The Parkinson’s Disease Questionnaire-39 (PDQ-39) was used to assess patients’ quality of life and social functioning.

### 2.3 MRI acquisition

MRI data were acquired at the Affiliated Brain Hospital of Nanjing Medical University using a 3.0-T Siemens Verio scanner (Siemens Verio, Germany). High-resolution structural images were obtained using a three-dimensional T1-weighted sequence with the following parameters: repetition time (TR) = 2000 ms, echo time (TE) = 3.34 ms, flip angle = 7°, 128 sagittal slices, slice thickness = 1.33 mm, and matrix size = 256 × 256. Resting-state functional MRI data were acquired using a T2-weighted echo-planar imaging sequence with 240 volumes. The acquisition parameters were as follows: TR = 2000 ms, TE = 30 ms, flip angle = 90°, field of view = 240 × 240 mm, matrix size = 64 × 64, 30 axial slices, slice thickness = 3.0 mm, and interslice gap = 0 mm. During scanning, participants were instructed to remain still, keep their eyes closed, stay awake, and avoid structured thinking.

### 2.4 Processing of rs-fMRI data

Resting-state fMRI data were preprocessed using SPM12 and related toolboxes implemented in MATLAB 2022b. Raw DICOM images were first converted to NIfTI format using dcm2niix, and image quality was visually checked using MRIcroGL. The first 10 volumes were discarded to allow for magnetization equilibrium and participant adaptation to the scanning environment. The remaining images underwent slice-timing correction and head-motion realignment. Participants with maximum head translation > 3 mm or rotation > 3° were excluded. Nuisance covariates including white matter signals, cerebrospinal fluid signals, and Friston-24 motion parameters were regressed out to reduce non-neuronal contributions. Functional images were then normalized to Montreal Neurological Institute (MNI) space using linear and nonlinear transformation and resampled to 3 × 3 × 3 mm^3^ voxels. Temporal band-pass filtering (0.01–0.08 Hz) was applied for resting-state signal analysis. Spatial smoothing with a 6-mm full width at half maximum (FWHM) Gaussian kernel was used for group-level ALFF analysis. For ReHo analysis, smoothing was performed after ReHo map calculation. Mean framewise displacement (FD) was calculated for each participant as an index of head motion and was compared across groups.

### 2.5 ALFF and ReHo calculation

Amplitude of low-frequency fluctuations (ALFF) was calculated to quantify the intensity of spontaneous low-frequency neural activity in each voxel during rest. For each participant, ALFF maps were generated from the preprocessed functional data and used for subsequent group-level analyses. ReHo was used to assess local synchronization of spontaneous neural activity. ReHo maps were calculated using Kendall’s coefficient of concordance (KCC), which reflects the temporal consistency between a given voxel and its neighboring voxels. Individual ReHo maps were subsequently normalized and spatially smoothed with a 6-mm FWHM Gaussian kernel for group analysis.

### 2.6 Whole-brain group analysis

Whole-brain statistical analyses were performed separately for ALFF and ReHo maps to identify regional abnormalities showing overall differences among the four PD subgroups. Specifically, one-way analysis of variance (ANOVA) was conducted across the PD-RBD^+^VH^+^, PD-RBD^+^VH^−^, PD-RBD^−^VH^+^, and PD-RBD^−^VH^−^ groups, with age, sex, disease duration, and LEDD included as covariates. For multiple-comparison correction, a voxel-level cluster-forming threshold of *p* < 0.001 was applied, and statistical significance was defined at cluster-level family-wise error (FWE)-corrected *p* < 0.05. For significant clusters, the peak brain region, MNI coordinates, peak *F* values, and cluster size were recorded. ROI values extracted from these clusters were z-transformed for visualization in subsequent figures. Mean values were extracted from these clusters for *post hoc* pairwise comparisons and subsequent region-constrained analyses. To examine whether the imaging findings were influenced by overall disease severity, additional whole-brain sensitivity analyses were performed by including total UPDRS as an extra covariate. The original model was retained as the primary model, whereas the UPDRS-adjusted model was used to evaluate the robustness of the imaging findings after accounting for overall clinical burden. The results of these sensitivity analyses are reported in Supplementary Table S5.

In addition, supplementary whole-brain voxel-wise 2 × 2 factorial analyses were performed for ALFF and ReHo maps using RBD status and VH status as two between-subject factors, with age, sex, disease duration, and LEDD as covariates. These supplementary factorial analyses were used to assess whether main or interaction effects could be detected at the voxel-wise whole-brain level after multiple-comparison correction.

### 2.7 ROI-based exploratory decomposition of RBD- and VH-related effects

To further characterize symptom-related patterns within brain regions showing significant overall between-group differences in the whole-brain four-group analyses, ROI values were extracted from the significant clusters identified in the whole-brain one-way ANOVA. These ROI values were then entered into a two-way ANOVA with a 2 × 2 factorial design in GraphPad Prism 9.0, with the presence or absence of RBD and VH treated as two fixed factors. This ROI-based analysis was intended to provide an exploratory decomposition of symptom-related effects within candidate regions, rather than a substitute for voxel-wise whole-brain confirmatory testing. Statistical results are reported as *F* values and corresponding *p* values, and partial eta squared (partial η^2^) was calculated as an estimate of effect size. Statistical significance was defined as *p* < 0.05. Because the ROIs were derived from whole-brain between-group effects in the same sample, these ROI-based factorial analyses were interpreted as *post hoc* candidate-region exploratory decomposition analyses rather than independent confirmatory tests. To account for multiple ROI-level tests, *p*-values from the ROI-based factorial analyses were adjusted using the Benjamini–Hochberg false discovery rate procedure across all tested ROI-level effects. Both nominal p values and FDR-corrected *q* values are reported. The FDR correction was applied across all 18 ROI-level tests, including RBD main effects, VH main effects, and RBD × VH interaction effects.

### 2.8 Statistical analysis

Demographic and clinical data were analyzed using SPSS 27.0. Continuous variables were expressed as mean ± standard deviation (SD) for normally distributed data and as median (interquartile range) for non-normally distributed data. Intergroup comparisons were performed using one-way ANOVA or the Kruskal–Wallis test, as appropriate. Categorical variables were presented as counts (percentages) and compared using *χ*^2^ tests. When overall group differences were significant, *post hoc* pairwise comparisons were performed using Tukey’s test for normally distributed variables and the corresponding non-parametric *post hoc* procedure for non-normally distributed variables, as appropriate. A two-sided *p* < 0.05 was considered statistically significant.

Additionally, to explore the clinical relevance of imaging abnormalities, ROI-based correlation analyses were performed between representative imaging metrics derived from significant clusters and clinical scale scores. These analyses were conducted in GraphPad Prism 9.0. Pearson correlation analysis was first used to examine associations between imaging measures and clinical variables. Significant correlations were subsequently re-evaluated using Spearman rank correlation analysis to test robustness. Correlation coefficients and corresponding *p*-values were reported. Multiple comparisons across correlation analyses were controlled using the Benjamini–Hochberg false discovery rate (FDR) procedure, and corrected q values were reported. Statistical significance after multiple testing was defined as *q* < 0.05.

## 3 Results

### 3.1 Demographic and clinical characteristics

This study enrolled 96 PD patients, who were divided into four groups based on the presence or absence of RBD and VH: PD-RBD^+^VH^+^ group, PD-RBD^+^VH^−^ group, PD-RBD^−^VH^+^ group and PD-RBD^−^VH^−^ group, 24 participants in each group. MRI and clinical scale data were collected from all subjects. Detailed demographic characteristics and clinical features are summarized in Table 1. No significant intergroup differences were observed in sex, age, BMI, disease duration, Hoehn and Yahr stage, or LEDD.

**Table 1:** Demographic and clinical characteristics of the four PD subgroups.

Variables	PD-RBD+VH+ (*n* = 24)	PD-RBD+VH– (*n* = 24)	PD-RBD–VH+ (*n* = 24)	PD-RBD–VH– (*n* = 24)	*p*-value
General information					
Sex (male, *N*%)	17 (70.8%)	12 (50.0%)	17 (70.8%)	13 (54.2%)	0.3099
Age (years)	64.9 ± 3.3	66.8 ± 6.5	63.3 ± 7.5	64.3 ± 6.4	0.2317
BMI (kg/m^2^)	24.4 ± 3.6	25.4 ± 2.1	24.9 ± 2.4	24.1 ± 2.5	0.4004
Disease duration (years)	4.5 ± 1.7	4.3 ± 1.4	4.7 ± 1.2	3.7 ± 1.4	0.0762
LEDD	764.4 ± 107.9	699.0 ± 82.9	749.0 ± 87.1	697.9 ± 152.0	0.0842
Assessment scales					
Hoehn and Yahr stage	2.3 ± 0.5	2.2 ± 0.6	2.4 ± 0.9	2.1 ± 0.6	0.5252
UPDRS (total)	45.5 ± 8.4	39.0 ± 7.7	42.0 ± 8.5	33.6 ± 6.2	<0.0001
UPDRS I	4.4 ± 1.3	2.6 ± 1.2	3.5 ± 1.6	1.8 ± 1.5	<0.0001
UPDRS II	9.1 ± 2.4	6.0 ± 1.4	8.5 ± 2.6	5.3 ± 2.6	<0.0001
UPDRS III	30.8 ± 8.0	29.8 ± 7.5	28.9 ± 6.8	25.9 ± 6.1	0.0991
UPDRS IV	1.2 ± 1.1	0.8 ± 0.7	1.0 ± 1.1	0.7 ± 0.8	0.2280
FOGQ	10.4 ± 3.4	7.9 ± 2.1	7.9 ± 2.0	2.3 ± 1.6	<0.0001
UM-PDHQ	7.3 ± 2.1	1.1 ± 0.8	6.0 ± 1.8	0.7 ± 0.7	<0.0001
RBDSQ	8.0 ± 1.9	7.2 ± 1.2	1.2 ± 1.2	0.8 ± 0.7	<0.0001
MoCA	22.00 (20.00, 25.75)	26.00 (24.00, 26.00)	25.00 (24.00, 26.00)	27.00 (26.00, 29.00)	<0.0001
PDQ39	49.3 ± 7.4	36.0 ± 14.6	36.2 ± 14.0	26.6 ± 11.0	<0.0001

*P*-values indicate overall group differences among the four subgroups. BMI, body mass index; LEDD, levodopa equivalent daily dose; UPDRS, Unified Parkinson’s Disease Rating Scale; FOGQ, Freezing of Gait Questionnaire; UM-PDHQ, University of Miami Parkinson’s Disease Hallucinations Questionnaire; RBDSQ, REM Sleep Behavior Disorder Screening Questionnaire; MoCA, Montreal Cognitive Assessment; PDQ-39, Parkinson’s Disease Questionnaire-39.

Significant differences were observed among the four groups in terms of total UPDRS score, UPDRS I, UPDRS II, FOGQ, UM-PDHQ, RBDSQ, MoCA, and PDQ-39 scores; however, no statistically significant differences were found among the groups in UPDRS III and UPDRS IV scores. Among the four groups, the PD-RBD^+^VH^+^ group showed the greatest overall clinical burden, with higher UPDRS total, FOGQ, UM-PDHQ, RBDSQ, and PDQ-39 scores and lower MoCA scores. The PD-RBD^−^VH^−^ group exhibited the mildest clinical symptoms. PD-RBD^+^VH^−^ group and PD-RBD^−^VH^+^ group exhibited intermediate patterns between the two groups. *Post hoc* pairwise comparisons further clarified the differential patterns among groups, and the specific results are shown in Supplementary Table S2. No significant between-group difference was observed in mean FD (Supplementary Table S1).

### 3.2 Abnormal brain regions in ALFF analysis

The ALFF analysis results demonstrated significant differences among the four groups in multiple brain regions (Table 2), including the left temporal pole, left cerebellar Crus I, right middle orbital frontal gyrus, left medial orbital frontal cortex (OFC), left middle frontal gyrus, left supplementary motor area (SMA). These regions were mainly distributed in temporal, frontal, cerebellar, and motor-related areas.

**Table 2:** Significant clusters identified by whole-brain ALFF analysis across the four PD subgroups.

Peak point brain region	MNI peak coordinates			*F* value	Cluster size
	*X*	*Y*	*Z*		
Left temporal pole	−42	15	−30	7.30	11
Left cerebellar Crus I	−27	−75	−24	6.02	14
Right middle orbital frontal gyrus	36	57	−15	6.60	14
Left medial orbital frontal gyrus	−12	48	−6	6.91	31
Left middle frontal gyrus	−39	39	30	6.97	15
Left supplementary motor area	−3	21	51	7.60	10

The table presents the peak brain regions, MNI coordinates, peak *F* values, and cluster sizes for brain areas showing significant between-group differences in ALFF. Coordinates are given in MNI space, and cluster size refers to the number of voxels within each significant cluster. ALFF, amplitude of low-frequency fluctuations; MNI, Montreal Neurological Institute; PD, Parkinson’s disease.

**Figure 1:**
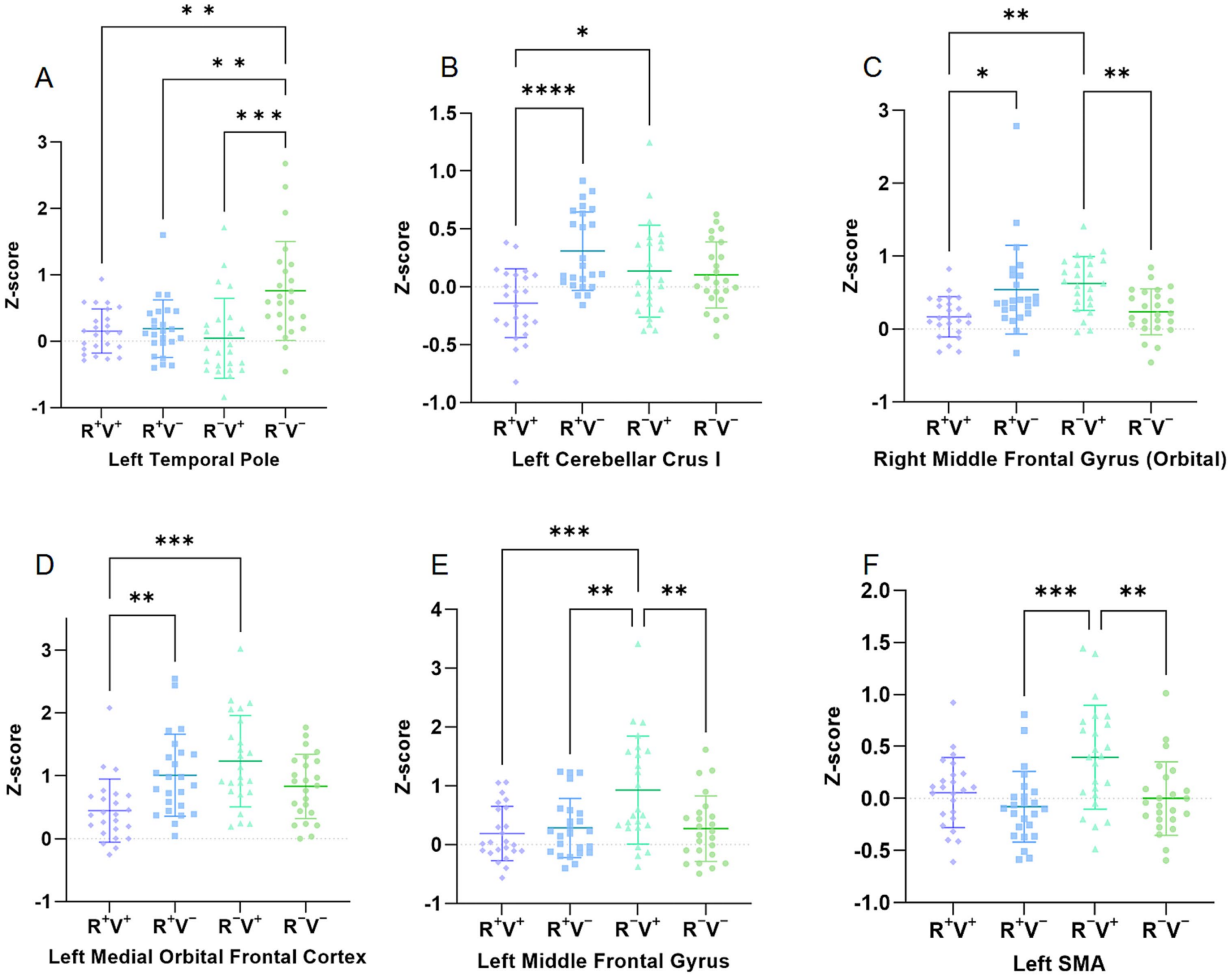
*Post hoc* comparisons of ALF*F* values among the four PD subgroups in brain regions showing significant group differences. Scatter plots illustrate the distribution of z-transformed ALFF values in the left temporal pole **(A)**, left cerebellar Crus I **(B)**, right middle orbital frontal gyrus **(C)**, left medial orbital frontal cortex **(D)**, left middle frontal gyrus **(E)**, and left supplementary motor area (SMA) **(F)** across the four subgroups stratified by rapid eye movement sleep behavior disorder (RBD) and visual hallucinations (VH). Each dot represents one participant. Error bars indicate mean ± standard deviation. Statistical significance for *post hoc* pairwise comparisons is indicated by brackets and asterisks (*p < 0.05, **p < 0.01, ***p < 0.001, ****p < 0.0001). ALFF, amplitude of low-frequency fluctuations; PD, Parkinson’s disease; SMA, supplementary motor area. Group labels: R^+^V^+^, PD-RBD^+^VH^+^; R^+^V^−^, PD-RBD^+^VH^−^; R^−^V^+^, PD-RBD^−^VH^+^; R^−^V^−^, PD-RBD^−^VH^−^.

*Post hoc* comparisons (Figure 1) showed that the PD-RBD^−^VH^−^ group exhibited higher ALFF values in certain regions, including the left temporal pole. Conversely, the PD-RBD^−^VH^+^ group exhibited relatively elevated ALFF in the right middle orbital frontal gyrus, left medial OFC, and left SMA. By comparison, PD-RBD^+^VH^+^ group exhibited relatively lower ALFF values in regions such as the left cerebellar Crus I and the left medial OFC. Additional whole-brain sensitivity analysis including total UPDRS as an extra covariate showed that the main ALFF findings remained detectable in regions overlapping with the left temporal pole, left cerebellar Crus I, right middle orbital frontal gyrus, left medial orbitofrontal cortex, left middle frontal gyrus, and left supplementary motor area, suggesting that these ALFF abnormalities were not solely explained by overall disease severity (Supplementary Table S5).

### 3.3 Abnormal brain regions in ReHo analysis

ReHo analysis revealed significant intergroup differences among the four PD groups across multiple brain regions, predominantly involving the cerebellum, frontal lobe, precentral gyrus, and precuneus (Table 3).

**Table 3:** Significant clusters identified by whole-brain ReHo analysis across the four PD subgroups.

Peak point brain region	MNI peak coordinates			*F* value	Cluster size
	*X*	*Y*	*Z*		
Left cerebellum VIII	−42	−39	−45	5.37	13
Left cerebellar Crus I	−54	−54	−30	7.05	19
Right middle orbital frontal gyrus	30	45	−15	6.90	11
Right inferior frontal gyrus, triangular part	39	21	27	7.41	23
Right precentral gyrus	39	−21	63	5.92	19
Left precuneus	−9	−57	78	8.11	18

The table presents the peak brain regions, MNI coordinates, peak *F* values, and cluster sizes for brain areas showing significant between-group differences in ReHo. Coordinates are given in MNI space, and cluster size refers to the number of voxels within each significant cluster. ReHo, regional homogeneity; MNI, Montreal Neurological Institute; PD, Parkinson’s disease.

**Figure 2:**
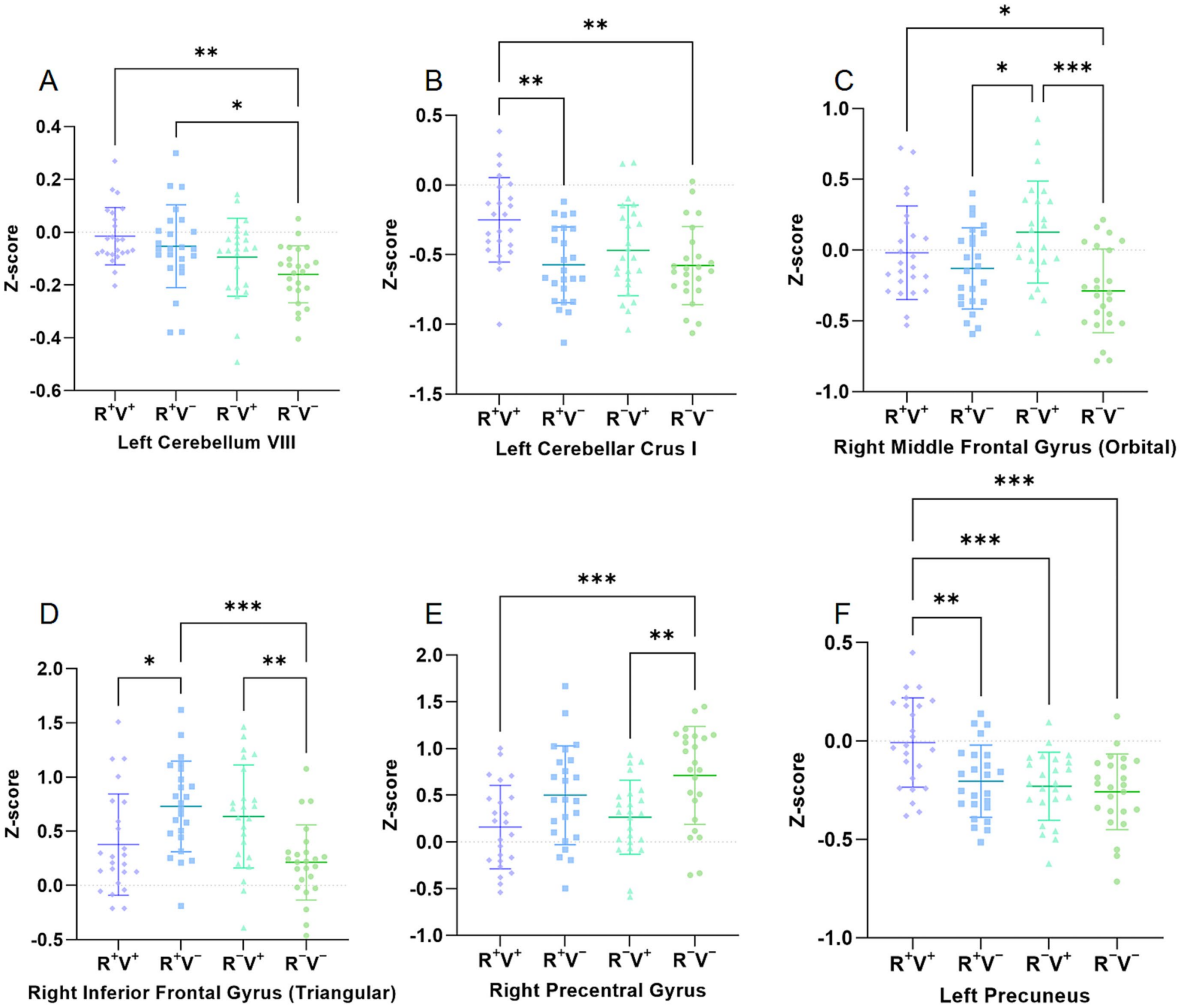
*Post hoc* comparisons of ReHo values among the four PD subgroups in brain regions showing significant group differences. Scatter plots illustrate the distribution of z-transformed ReHo values in the left cerebellum VIII **(A)**, left cerebellar Crus I **(B)**, right middle orbital frontal gyrus **(C)**, right inferior frontal gyrus, triangular part **(D)**, right precentral gyrus **(E)**, and left precuneus **(F)** across the four subgroups stratified by rapid eye movement sleep behavior disorder (RBD) and visual hallucinations (VH). Each dot represents one participant. Error bars indicate mean ± standard deviation. Statistical significance for *post hoc* pairwise comparisons is indicated by brackets and asterisks (*p < 0.05, **p < 0.01, ***p < 0.001). ReHo, regional homogeneity; PD, Parkinson’s disease. Group labels: R^+^V^+^, PD-RBD^+^VH^+^; R^+^V^−^, PD-RBD^+^VH^−^; R^−^V^+^, PD-RBD^−^VH^+^; R^−^V^−^, PD-RBD^−^VH^−^.

*Post hoc* comparisons showed region-specific subgroup patterns (Figure 2). In the left cerebellar lobule VIII, the PD-RBD^−^VH^−^ group showed lower ReHo values than the PD-RBD^+^VH^+^ and PD-RBD^+^VH^−^ groups. In the left cerebellar Crus I, the PD-RBD^+^VH^+^ group showed higher ReHo values than the PD-RBD^+^VH^−^ and PD-RBD^−^VH^−^ groups. In the right middle orbital frontal gyrus, the PD-RBD^−^VH^+^ group showed the highest ReHo values, whereas the PD-RBD^−^VH^−^ group showed the lowest values. In the right inferior frontal gyrus, triangular part, the PD-RBD^−^VH^−^ group showed relatively lower ReHo values than the PD-RBD^+^VH^−^ and PD-RBD^−^VH^+^ groups. In the right precentral gyrus, the PD-RBD^−^VH^−^ group showed higher ReHo values than the PD-RBD^+^VH^+^ and PD-RBD^−^VH^+^ groups. In the left precuneus, the PD-RBD^+^VH^+^ group showed higher ReHo values than the other three subgroups. These findings indicate heterogeneous regional ReHo abnormalities across PD subgroups stratified by RBD and VH status. Additional UPDRS-adjusted whole-brain sensitivity analysis for ReHo showed residual group differences in the right precentral gyrus, right inferior frontal gyrus triangular part, right middle orbital frontal gyrus, left precuneus, and left cerebellar Crus I, indicating that several ReHo abnormalities remained detectable after accounting for overall disease severity, although some ReHo effects were attenuated (Supplementary Table S5).

### 3.4 Exploratory ROI-based factorial analysis within candidate regions

To further characterize symptom-related patterns within regions showing significant overall between-group differences, we performed *post hoc* candidate-region exploratory decomposition analyses using a 2 × 2 factorial framework (Table 4). After Benjamini–Hochberg FDR correction across all ROI-level tests, RBD-related candidate-region patterns survived correction in precuneus ReHo, cerebellar lobule VIII ReHo, and SMA ALFF. VH-related candidate-region patterns survived correction in OFC ReHo, precuneus ReHo, cerebellar Crus I ReHo, SMA ALFF, and temporal pole ALFF. Interaction-like patterns survived correction in OFC ReHo and temporal pole ALFF, whereas the precuneus interaction did not survive FDR correction (Supplementary Table S6). Because these ROIs were derived from the same whole-brain four-group analysis, these findings should be interpreted as *post hoc* exploratory candidate-region results rather than independent confirmatory evidence.

**Table 4:** *Post hoc* exploratory candidate-region decomposition of imaging measures according to RBD and VH status.

ROI	RBD main effect			VH main effect			RBD × VH interaction		
	*F*	*P*	Partial *η*^2^	*F*	*P*	Partial *η*^2^	*F*	*P*	Partial *η*^2^
OFC ReHo	*F*(1, 92) = 0.01	0.9215	0.0001	*F*(1, 92) = 16.24	0.0001	0.1500	*F*(1, 92) = 5.47	0.0216	0.0561
Precuneus ReHo	*F*(1, 92) = 11.99	0.0008	0.1153	*F*(1, 92) = 7.99	0.0058	0.0799	*F*(1, 92) = 4.48	0.0371	0.0464
Cerebellum VIII ReHo	*F*(1, 92) = 11.90	0.0009	0.1145	*F*(1, 92) = 3.61	0.0607	0.0377	*F*(1, 92) = 0.25	0.6192	0.0027
Cerebellum Crus I ReHo	*F*(1, 92) = 3.49	0.0649	0.0366	*F*(1, 92) = 12.82	0.0006	0.1223	*F*(1, 92) = 3.17	0.0784	0.0333
SMA ALFF	*F*(1, 92) = 6.99	0.0096	0.0706	*F*(1, 92) = 11.30	0.0011	0.1094	*F*(1, 92) = 2.71	0.1031	0.0286
Temporal pole ALFF	*F*(1, 92) = 4.16	0.0443	0.0433	*F*(1, 92) = 11.09	0.0013	0.1076	*F*(1, 92) = 9.09	0.0033	0.0899

The table summarizes the results of the factorial analysis for selected ROIs, including the main effects of RBD and VH and their interaction. For each effect, *F* statistics, corresponding *P*-values, and effect sizes (partial *η*^2^) are reported. Selected ROIs were derived from regions showing significant alterations in the whole-brain ALFF or ReHo analyses. ROI, region of interest; PD, Parkinson’s disease; RBD, rapid eye movement sleep behavior disorder; VH, visual hallucinations; OFC, orbitofrontal cortex; ReHo, regional homogeneity; ALFF, amplitude of low-frequency fluctuations; SMA, supplementary motor area.

### 3.5 Clinical correlation analysis

**Figure 3:**
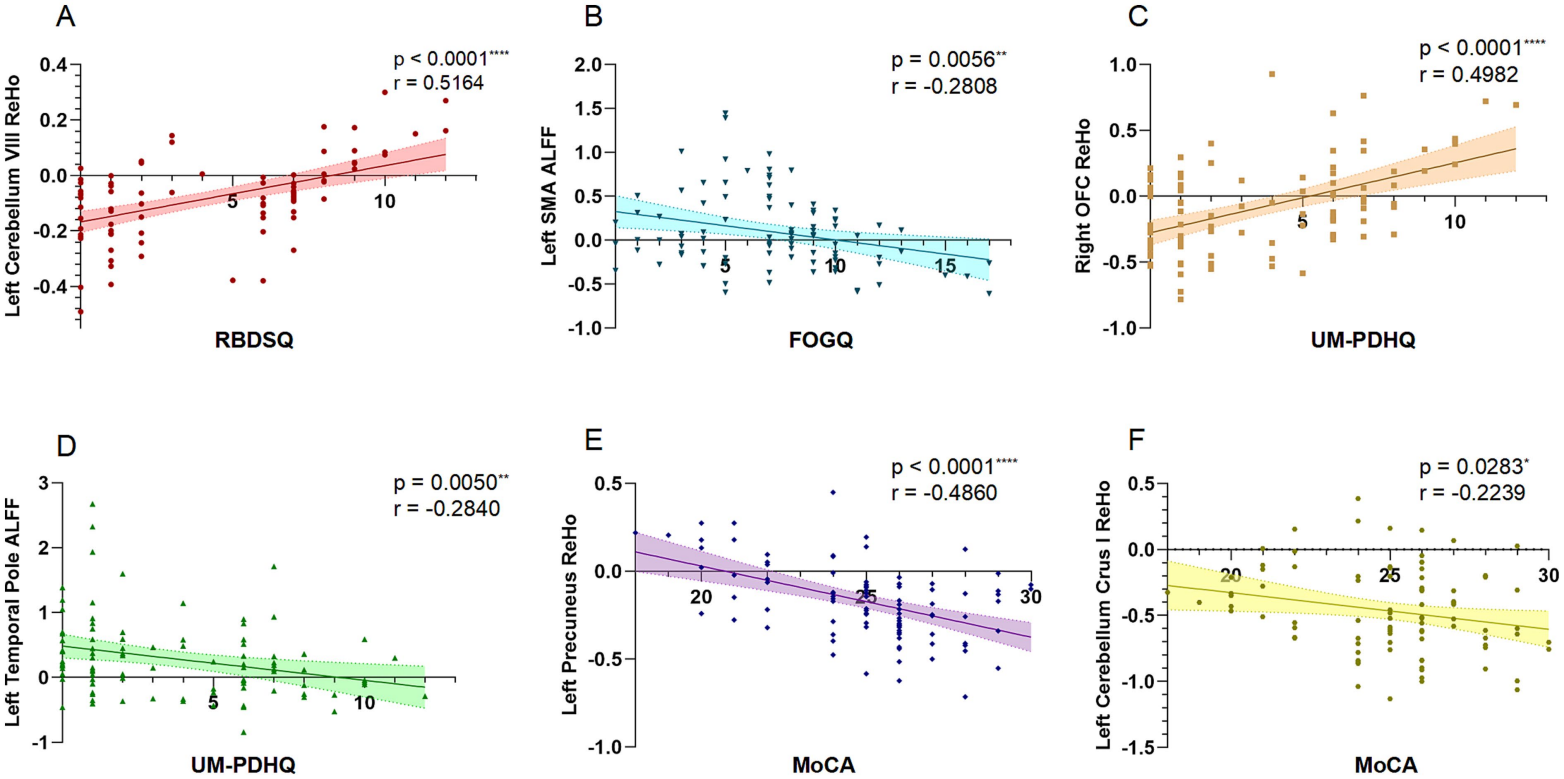
Correlations between imaging metrics and clinical scale scores in patients with Parkinson’s disease. Scatter plots show the associations between regional imaging measures and clinical scales. **(A)** Left cerebellum VIII ReHo was positively correlated with RBDSQ score. **(B)** Left SMA ALFF was negatively correlated with FOGQ score. **(C)** Right orbital frontal cortex (OFC) ReHo was positively correlated with UM-PDHQ score. **(D)** Left temporal pole ALFF was negatively correlated with UM-PDHQ score. **(E)** Left precuneus ReHo was negatively correlated with MoCA score. **(F)** Left cerebellar Crus I ReHo showed a negative association with MoCA score in the Pearson analysis, but this association did not survive Spearman-based FDR correction and was therefore interpreted as exploratory. Solid lines represent the fitted regression lines and shaded areas indicate 95% confidence intervals. ALFF, amplitude of low-frequency fluctuations; ReHo, regional homogeneity; SMA, supplementary motor area; OFC, orbitofrontal cortex; RBDSQ, REM Sleep Behavior Disorder Screening Questionnaire; FOGQ, Freezing of Gait Questionnaire; UM-PDHQ, University of Miami Parkinson’s Disease Hallucinations Questionnaire; MoCA, Montreal Cognitive Assessment.

To further explore the relationship between local brain dysfunction and clinical symptoms, we first conducted Pearson correlation analyses on imaging metrics and clinical scale scores of brain regions with significant intergroup differences (Figure 3).

Left cerebellum VIII ReHo was positively correlated with RBDSQ scores, indicating that higher local synchrony in this region was associated with more severe RBD symptoms (Figure 3A). The left SMA ALFF showed a significant negative correlation with the FOGQ score, indicating that lower spontaneous neural activity in the SMA correlates with greater freezing of gait severity (Figure 3B).

Analysis related to VH revealed a significant positive correlation between ReHo in the right OFC and UM-PDHQ scores, while a significant negative correlation was observed between ALFF in the left temporal pole and UM-PDHQ scores. These findings suggest that enhanced local synchronicity in the orbitofrontal cortex and reduced spontaneous activity in the temporal pole are both associated with increased hallucination burden (Figures 3C,D).

In the cognitive correlation analysis, left precuneus ReHo was negatively correlated with MoCA scores and remained significant after Spearman rank correlation analysis and FDR correction. Left cerebellar Crus I ReHo showed a negative association with MoCA scores in the Pearson analysis, but this association did not remain significant after Spearman analysis and FDR correction; therefore, it was interpreted cautiously as an exploratory correlation (Figures 3E,F and Supplementary Table S3).

Among the six reported clinical correlations, five remained significant after Spearman rank correlation analysis and Benjamini–Hochberg FDR correction. The left cerebellar Crus I ReHo–MoCA association showed the same direction but did not survive FDR correction (Supplementary Table S3).

## 4 Discussion

This study first identified regional abnormalities across four clinically defined PD subgroups using whole-brain four-group analyses, and then performed exploratory ROI-based factorial analyses to further characterize the relative contributions of RBD and VH within candidate regions. Patients in the PD-RBD^+^VH^+^ group exhibited the greatest clinical burden and poorer cognitive status, suggesting that the comorbidity of RBD and VH may correspond to a more severe clinical subtype. Analyses integrating ALFF and ReHo revealed regional abnormalities involving cerebellar, motor, frontal-association, and precuneus-related regions. Additional voxel-wise whole-brain 2 × 2 factorial analyses did not reveal corrected main or interaction effects, indicating that the present symptom-related decomposition should be interpreted primarily at the candidate-region exploratory level rather than as definitive whole-brain factorial evidence. Meanwhile, the significant correlations between neuroimaging indices and clinical scale scores further supported the clinical relevance of these regional functional abnormalities.

A notable finding was that the PD-RBD^+^VH^+^ group exhibited the greatest clinical burden, characterized by more severe non-motor symptoms, worse cognitive performance, and greater gait-related impairment. It is consistent with previous evidence that both RBD and VH are associated with unfavorable progression and poorer prognosis in PD (5–7). More importantly, our findings suggest that the coexistence of these two symptoms may define a subgroup with more widespread system-level dysfunction, rather than simply reflecting the additive effect of two non-motor manifestations. Such a phenotype is unlikely to be explained by nigrostriatal dopaminergic degeneration alone (9), but may reflect broader dysfunction involving cortical–subcortical integration, brainstem ascending systems, and non-dopaminergic pathways (6, 28, 29). Clinically, this implies that the coexistence of RBD and VH may serve as a critical clue for identifying high-risk patients and optimizing symptom stratification.

From the perspective of the candidate-region exploratory analysis, RBD-related abnormalities were primarily concentrated in brain regions involved in sensorimotor integration, motor automaticity, and postural-gait regulation. Cerebellar lobule VIII ReHo showed an FDR-corrected RBD-related candidate-region pattern and was positively correlated with RBDSQ scores, suggesting that greater local synchrony abnormalities in cerebellar regions associated with motor regulation were accompanied by more severe RBD symptoms. Cerebellar lobule VIII is closely involved in sensorimotor integration, postural control, and motor coordination (30, 31). Therefore, this result suggests that RBD-related brain dysfunction may not be limited to sleep behavior abnormalities themselves, but may further affect cerebellar networks associated with motor automation and central axis control (32, 33). In addition, SMA ALFF showed an FDR-corrected RBD-related candidate-region pattern and was negatively correlated with the FOGQ score, suggesting that reduced local spontaneous activity is closely associated with more severe freezing of gait (34, 35). Overall, this study suggests that the functional abnormalities corresponding to RBD in PD may extend from the sleep-brainstem system to the cerebellum-SMA-sensory-motor network, which also provides exploratory imaging evidence related to more pronounced postural and gait disturbances commonly observed in RBD patients (32, 36).

In contrast, VH-related abnormalities were more prominently distributed in regions associated with reality monitoring, semantic integration, and higher-order perceptual processing. The analysis showed that OFC ReHo showed an FDR-corrected VH-related candidate-region pattern and was positively correlated with hallucination severity, suggesting that enhanced local synchrony abnormalities were associated with a greater burden of hallucinatory symptoms. The OFC plays a key role in reality testing, correction of misattribution errors, and top-down regulation (37–39), its functional impairment may weaken patients’ ability to distinguish internally generated representations from externally derived perceptual input, thereby facilitating the emergence of hallucinations. Meanwhile, ALFF in the left temporal pole was negatively correlated with UM-PDHQ scores, indicating that reduced spontaneous activity in this region was associated with greater hallucination severity. Given that the temporal pole is involved in semantic processing, contextual attribution, and multimodal information integration (40, 41), these findings suggest that the occurrence of VH may arise not only from abnormalities in primary perceptual input, but also from erroneous assignment of meaning during higher-order associative and interpretive processing (42, 43). Overall, the brain function alterations associated with VH in this study suggest that frontal lobe-associative cortex network imbalance may play a significant role in higher-order perceptual abnormalities.

An important observation of the present study is that exploratory ROI-based analyses identified significant RBD × VH interaction patterns in the OFC, and temporal pole, whereas the precuneus interaction did not survive FDR correction. These findings should be interpreted within the context of candidate-region analyses, but they nevertheless suggest that the coexistence of RBD and VH may be associated with non-additive disruption of higher-order integrative systems in PD. The OFC and temporal pole are involved in reality monitoring, semantic integration, and higher-order perceptual interpretation, suggesting that the coexistence of RBD and VH may be associated with altered function in regions supporting perceptual and associative processing. Although the precuneus interaction did not survive FDR correction, its trend-level interaction pattern, FDR-corrected RBD- and VH-related patterns, and robust association with MoCA suggest that this region may still be clinically relevant to cognitive vulnerability in PD patients with coexisting RBD and VH (44–46). Although the Crus I–MoCA association did not survive Spearman-based FDR correction, it showed a negative direction in the initial Pearson analysis and therefore may still provide exploratory information regarding cerebellar involvement in cognitive vulnerability. This interpretation is supported by the FDR-corrected VH-related pattern in Crus I ReHo and by the persistence of Crus I ReHo abnormalities in the UPDRS-adjusted sensitivity analysis. Given the established role of Crus I in cerebellar cognitive circuits, the present findings suggest that Crus I may represent a candidate cerebellar region linking hallucination-related clinical status with cognitive dysfunction in PD (47–49). However, this association should be interpreted cautiously and requires validation in larger samples with more comprehensive cognitive assessment.

Equally noteworthy, the SMA findings may indicate a potential link between non-motor symptom status and axial motor dysfunction in PD. The SMA is responsible for the initiation and termination of motor sequences, generation of internal rhythm, and execution of motor planning, and its dysfunction has been widely recognized as being closely associated with gait initiation failure, impaired rhythm maintenance, and axial motor symptoms in patients with PD (34, 50, 51). In the present study, SMA ALFF showed FDR-corrected RBD- and VH-related candidate-region patterns and was negatively associated with freezing of gait severity, suggesting that altered SMA activity may be linked to axial motor burden in PD patients with RBD- or VH-related clinical features. This finding suggests that, within the complex clinical phenotype of PD, non-motor symptoms and motor dysfunction may be partly associated with overlapping neural systems, rather than representing entirely independent processes (52–54).

Supplementary whole-brain voxel-wise 2 × 2 factorial analyses were additionally performed for both ALFF and ReHo using RBD status and VH status as two factors. No main effect of RBD, main effect of VH, or RBD × VH interaction survived whole-brain multiple-comparison correction for either ALFF or ReHo. In the ReHo analysis, uncorrected exploratory clusters were observed for the RBD × VH interaction at a voxel-wise threshold of *p* < 0.005 with cluster size > 10 voxels; however, these findings did not survive whole-brain multiple-comparison correction and were therefore interpreted cautiously as exploratory voxel-wise signals only.

Several limitations should be acknowledged. First, RBD status was based on clinical history, family report, and RBDSQ score rather than polysomnography-confirmed diagnosis; therefore, participants were classified as probable RBD, and potential misclassification cannot be excluded. Similarly, VH status was determined using clinical interview and UM-PDHQ assessment rather than a formal structured psychiatric diagnostic interview, which may also introduce classification uncertainty. Second, although the primary whole-brain analyses adjusted for age, sex, disease duration, and LEDD, the four groups differed in several clinical variables, including total UPDRS, FOGQ, MoCA, and PDQ-39. Additional sensitivity analyses adjusting for total UPDRS suggested that several findings were not solely explained by overall disease severity, but residual confounding by cognitive status, quality of life, gait burden, or other unmeasured clinical factors remains possible. Third, although FDR correction was applied across ROI-level factorial tests, the ROIs were selected from the same whole-brain four-group analysis. Therefore, these ROI-based decomposition results may still be affected by selection bias and should be interpreted as *post hoc* exploratory candidate-region findings rather than independent confirmatory evidence.

In conclusion, PD patients with coexisting RBD and VH showed greater clinical burden and regional functional abnormalities involving cerebellar, frontal-association, motor, temporal, and precuneus-related regions. Additional UPDRS-adjusted sensitivity analyses suggested that several abnormalities were not solely explained by overall disease severity, although some effects were attenuated. The ROI-based decomposition findings should be interpreted as exploratory and hypothesis-generating rather than confirmatory evidence of independent RBD, VH, or interaction effects. Larger longitudinal studies with polysomnography-confirmed RBD, structured psychiatric assessment of VH, and multimodal imaging are needed to validate these candidate regional abnormalities and clarify their clinical relevance.

## Data Availability

The raw data supporting the conclusions of this article will be made available by the corresponding authors upon reasonable request.
