# Supplementary material for: Regional brain dysfunction patterns associated with rapid eye movement sleep behavior disorder and visual hallucinations in Parkinson’s disease: a resting-state fMRI study with exploratory ROI-based factorial analysis
Source: Front Neurol. 2026 Jun 24;17:1858348. doi: 10.3389/fneur.2026.1858348 (PMC13341548; doi:10.3389/fneur.2026.1858348)
Supplement: Supplementary file 1 [file Table_1.DOCX]

Supplementary Material

# Supplementary Tables

**Supplementary Table S1.** Mean framewise displacement (FD) across the four PD subgroups.

|  | **PD-RBD^+^VH^+^**  **(n = 24)** | **PD-RBD^+^VH^–^**  **(n = 24)** | **PD-RBD^–^VH^+^**  **(n = 24)** | **PD-RBD^–^VH^–^**  **(n = 24)** | ***p*-value** |
| --- | --- | --- | --- | --- | --- |
| Mean FD | 0.11 (0.07, 0.17) | 0.15 (0.10, 0.25) | 0.14 (0.08, 0.23) | 0.15 (0.10, 0.28) | 0.1938 |

Mean FD was calculated for each participant as an index of head motion during rs-fMRI acquisition. Values are presented as median (interquartile range). P values indicate overall between-group differences among the four subgroups. Abbreviations: FD, framewise displacement; PD, Parkinson’s disease; RBD, rapid eye movement sleep behavior disorder; VH, visual hallucinations.

**Supplementary Table S2.** Post hoc analyses of demographic and clinical variables among the four PD subgroups.

|  | ***p*-value (post hoc)** | | | | | |
| --- | --- | --- | --- | --- | --- | --- |
|  | **G1 VS G2** | **G1 VS G3** | **G1 VS G4** | **G2 VS G3** | **G2 VS G4** | **G3 VS G4** |
| Sex (male, N%) | 0.4536 | >0.9999 | 0.6384 | 0.4536 | 0.9909 | 0.6384 |
| Age (years) | 0.6991 | 0.7812 | 0.9847 | 0.1846 | 0.4781 | 0.9349 |
| BMI (kg/m2) | 0.6436 | 0.9246 | 0.9731 | 0.9454 | 0.3802 | 0.7188 |
| Disease duration (years) | 0.9718 | 0.9497 | 0.1993 | 0.7640 | 0.4111 | 0.0623 |
| LEDD | 0.1797 | 0.9630 | 0.1686 | 0.4053 | >0.9999 | 0.3868 |
| Hoehn & Yahr stage | 0.8147 | 0.9962 | 0.7548 | 0.6897 | 0.9995 | 0.6213 |
| UPDRS (total) | 0.0236 | 0.3841 | <0.0001 | 0.5641 | 0.0774 | 0.0018 |
| UPDRS I | 0.0001 | 0.1151 | <0.0001 | 0.1151 | 0.2140 | 0.0003 |
| UPDRS II | <0.0001 | 0.7802 | <0.0001 | 0.0012 | 0.7074 | <0.0001 |
| UPDRS III | 0.9575 | 0.7882 | 0.0825 | 0.9741 | 0.2341 | 0.4551 |
| UPDRS IV | 0.4361 | 0.9689 | 0.2747 | 0.7166 | 0.9904 | 0.5285 |
| FOGQ | 0.0027 | 0.0033 | <0.0001 | >0.9999 | <0.0001 | <0.0001 |
| UM-PDHQ | <0.0001 | 0.0170 | <0.0001 | <0.0001 | 0.7647 | <0.0001 |
| RBDSQ | 0.1598 | <0.0001 | <0.0001 | <0.0001 | <0.0001 | 0.6173 |
| MoCA | 0.0006 | 0.0113 | <0.0001 | 0.8086 | 0.0113 | 0.0006 |
| PDQ39 | 0.0015 | 0.0017 | <0.0001 | >0.9999 | 0.0400 | 0.0353 |

The table summarizes the adjusted P values from post hoc pairwise comparisons of demographic features and clinical assessment scores among the four PD subgroups stratified by RBD and VH. G1, PD-RBD^+^VH^+^; G2, PD-RBD^+^VH^−^; G3, PD-RBD^−^VH^+^; G4, PD-RBD^−^VH^−^. Abbreviations: BMI, body mass index; LEDD, levodopa equivalent daily dose; UPDRS, Unified Parkinson’s Disease Rating Scale; FOGQ, Freezing of Gait Questionnaire; UM-PDHQ, University of Miami Parkinson’s Disease Hallucinations Questionnaire; RBDSQ, REM Sleep Behavior Disorder Screening Questionnaire; MoCA, Montreal Cognitive Assessment; PDQ-39, Parkinson’s Disease Questionnaire-39.

**Supplementary Table S3.** Sensitivity analysis of correlations between neuroimaging indices and clinical measures using Spearman correlation and FDR correction.

| **ROI** | **Clinical scale** | **Spearman ρ** | **Raw P** | **FDR-corrected q value** | **Survived FDR correction** |
| --- | --- | --- | --- | --- | --- |
| Cerebellum VIII ReHo | RBDSQ | 0.4937 | < 0.0001 | ≤ 0.0002 | Yes |
| SMA ALFF | FOGQ | -0.2568 | 0.0115 | 0.0138 | Yes |
| OFC ReHo | UM-PDHQ | 0.4411 | < 0.0001 | ≤ 0.0002 | Yes |
| Temporal pole ALFF | UM-PDHQ | -0.2890 | 0.0043 | 0.0065 | Yes |
| Precuneus ReHo | MoCA | -0.4462 | < 0.0001 | ≤ 0.0002 | Yes |
| Cerebellum Crus I ReHo | MoCA | -0.1852 | 0.0709 | 0.0709 | No |

To further ensure the robustness of the observed associations, all correlations originally assessed using Pearson analysis were re-tested using Spearman rank correlation, and the resulting P values were additionally corrected using the false discovery rate (FDR) method. The table reports Spearman’s ρ, raw P values, FDR-corrected q values, and whether each association remained significant after FDR correction. Abbreviations: ROI, region of interest; FDR, false discovery rate; ReHo, regional homogeneity; ALFF, amplitude of low-frequency fluctuations; SMA, supplementary motor area; OFC, orbitofrontal cortex.

**Supplementary Table S4.** Uncorrected exploratory clusters identified in the supplementary whole-brain voxel-wise 2 × 2 factorial ReHo analysis.

| **Peak point brain region** | **X** | **Y** | **Z** | **F value** | **Cluster Size** |
| --- | --- | --- | --- | --- | --- |
| Right inferior occipital gyrus | 42 | -87 | -12 | 13.88 | 10 |
| Right middle frontal gyrus | 33 | 57 | 21 | 13.80 | 14 |

The table summarizes clusters observed for the RBD × VH interaction at an exploratory uncorrected threshold (voxel-wise p < 0.005, cluster size > 10 voxels). No main effect of RBD, main effect of VH, or RBD × VH interaction survived whole-brain multiple-comparison correction for either ALFF or ReHo. Therefore, the clusters shown here are provided for exploratory reference only and should be interpreted with caution. Coordinates are reported in MNI space.

**Supplementary Table S5.** UPDRS-adjusted whole-brain sensitivity analysis of ALFF and ReHo abnormalities.

| **Metric** | **Brain region** | **x** | **y** | **z** | **Peak F** | **Cluster size** | **P-FWE** |
| --- | --- | --- | --- | --- | --- | --- | --- |
| zALFF | Left temporal pole | -42 | 15 | -30 | 7.2250 | 11 | 0.0046 |
| zALFF | Left cerebellar Crus I | -27 | -75 | -24 | 5.8087 | 14 | 0.0244 |
| zALFF | Right middle orbital frontal gyrus | 30 | 63 | -12 | 9.1051 | 33 | 0.0005 |
| zALFF | Left medial orbitofrontal cortex | -12 | 48 | -6 | 7.1158 | 33 | 0.0052 |
| zALFF | Left middle frontal gyrus | -39 | 39 | 30 | 7.1565 | 15 | 0.0050 |
| zALFF | Left supplementary motor area | -3 | 21 | 51 | 7.8416 | 11 | 0.0023 |
| zKccReHo | Right precentral gyrus | 36 | -18 | 63 | 6.9610 | 10 | 0.0020 |
| zKccReHo | Right inferior frontal gyrus, triangular part | 36 | 18 | 27 | 6.4202 | 18 | 0.0038 |
| zKccReHo | Right middle orbital frontal gyrus | 33 | 45 | -15 | 6.4135 | 6 | 0.0038 |
| zKccReHo | Left precuneus | -9 | -54 | 75 | 9.1803 | 9 | 0.0002 |
| zKccReHo | Left cerebellar Crus I | -51 | -54 | -30 | 7.0353 | 8 | 0.0018 |

Whole-brain sensitivity analyses were performed by including total UPDRS score as an additional covariate to account for overall disease severity. Only regions overlapping with the main text findings are listed. Coordinates are reported in Montreal Neurological Institute space. Peak F indicates the maximum F value within each cluster. P-FWE values indicate family-wise error–corrected significance. ALFF, amplitude of low-frequency fluctuations; ReHo, regional homogeneity; UPDRS, Unified Parkinson’s Disease Rating Scale; SMA, supplementary motor area.

**Supplementary Table S6.** FDR-corrected exploratory ROI-based factorial analysis within candidate regions.

| **ROI** | **Effect** | **P** | **FDR q** |
| --- | --- | --- | --- |
| OFC ReHo | RBD main effect | 0.9215 | 0.9215 |
| OFC ReHo | VH main effect | 0.0001 | 0.0018 |
| OFC ReHo | RBD × VH interaction | 0.0216 | 0.0389 |
| Precuneus ReHo | RBD main effect | 0.0008 | 0.0039 |
| Precuneus ReHo | VH main effect | 0.0058 | 0.0131 |
| Precuneus ReHo | RBD × VH interaction | 0.0371 | 0.0607 |
| Cerebellum VIII ReHo | RBD main effect | 0.0009 | 0.0039 |
| Cerebellum VIII ReHo | VH main effect | 0.0607 | 0.0834 |
| Cerebellum VIII ReHo | RBD × VH interaction | 0.6192 | 0.6556 |
| Cerebellum Crus I ReHo | RBD main effect | 0.0649 | 0.0834 |
| Cerebellum Crus I ReHo | VH main effect | 0.0006 | 0.0039 |
| Cerebellum Crus I ReHo | RBD × VH interaction | 0.0784 | 0.0941 |
| SMA ALFF | RBD main effect | 0.0096 | 0.0192 |
| SMA ALFF | VH main effect | 0.0011 | 0.0039 |
| SMA ALFF | RBD × VH interaction | 0.1031 | 0.1160 |
| Temporal pole ALFF | RBD main effect | 0.0443 | 0.0665 |
| Temporal pole ALFF | VH main effect | 0.0013 | 0.0039 |
| Temporal pole ALFF | RBD × VH interaction | 0.0033 | 0.0085 |

ROI-based 2 × 2 factorial analyses were performed within candidate regions derived from the whole-brain four-group analysis to explore RBD-related, VH-related, and RBD × VH interaction-like patterns. Nominal P values are shown together with Benjamini–Hochberg FDR-corrected q values. FDR correction was applied across all ROI-level tests, including RBD main effects, VH main effects, and RBD × VH interaction effects. These analyses were interpreted as post hoc exploratory candidate-region findings rather than independent confirmatory evidence, because the ROIs were derived from the same whole-brain analysis. ROI, region of interest; RBD, rapid eye movement sleep behavior disorder; VH, visual hallucinations; ALFF, amplitude of low-frequency fluctuations; ReHo, regional homogeneity; OFC, orbitofrontal cortex; SMA, supplementary motor area; FDR, false discovery rate.
